# Targeted misexpression of *NAC052*, acting in H3K4 demethylation, alters leaf morphological and anatomical traits in *Arabidopsis thaliana*

**DOI:** 10.1093/jxb/erz509

**Published:** 2019-11-19

**Authors:** Roxanne van Rooijen, Stefanie Schulze, Patrick Petzsch, Peter Westhoff

**Affiliations:** 1 Institute of Plant Molecular and Developmental Biology, Heinrich-Heine-University, Duesseldorf, Germany; 2 Cluster of Excellence on Plant Sciences ‘From Complex Traits towards Synthetic Modules’, Duesseldorf, Germany; 3 Biologisch-Medizinisches Forschungszentrum (BMFZ), Genomics & Transcriptomics Labor (GTL), Heinrich-Heine-University, Duesseldorf, Germany; 4 University of Cambridge, UK

**Keywords:** Activation tagging, C_4_ photosynthesis, histone modifications, Kranz anatomy, leaf development, NAC052

## Abstract

In an effort to identify genetic regulators for the cell ontogeny around the veins in *Arabidopsis thaliana* leaves, an activation-tagged mutant line with altered leaf morphology and altered bundle sheath anatomy was characterized. This mutant had a small rosette area with wrinkled leaves and chlorotic leaf edges, as well as enhanced chloroplast numbers in the (pre-)bundle sheath tissue. It had a bundle-specific promoter from the gene *GLYCINE DECARBOXYLASE SUBUNIT-T* from the C_4_ species *Flaveria trinervia* (*GLDT*_*Ft*_ promoter) inserted in the coding region of the transcriptional repressor *NAC052*, functioning in H3K4 demethylation, in front of an alternative start codon in-frame with the natural start codon. Reconstruction of the mutation event of our activation-tagged line by creating a line expressing an N-terminally truncated sequence of NAC052 under control of the *GLDT*_*Ft*_ promoter confirmed the involvement of *NAC052* in leaf development. Our study not only reveals leaf anatomic and transcriptomic effects of an N-terminally truncated NAC052 under control of the *GLDT*_*Ft*_ promoter, but also identifies *NAC052* as a novel genetic regulator of leaf development.

## Teaser

This study identifies the transcriptional repressor NAC052, acting in H3K4 demethylation, as a regulator of leaf anatomical and morphological traits.

## Introduction

In C_4_ photosynthesis, the mesophyll cells of the leaf fix incoming CO_2_ into a C_4_ acid. Subsequently, the C_4_ acid is transported into specialized bundle sheath (BS) cells where it becomes decarboxylated. The CO_2_ that is released during the decarboxylation of the C_4_ acid is concentrated around the enzyme Rubisco. The increased concentration of CO_2_ around Rubisco reduces the rate of photorespiration, leading to enhanced photosynthetic efficiency particularly in hot and/or dry environments (Sage, 2004; Zhu *et al.*, 2010). In terms of leaf anatomy, the BS cells in C_4_ plants are organized around the leaf veins in a wreath-like structure called Kranz anatomy (Haberlandt, 1896). The BS cells are interconnected with the mesophyll cells through plasmodesmata (Hatch, 1987). Compared with mesophyll cells, C_4_ BS cells are enlarged and enriched with chloroplasts (Welkie and Caldwell, 1970; Dengler and Nelson, 1999).

C_4_ photosynthesis has evolved at least 60 times independently (Sage *et al.*, 2012). Because of these multiple independent occurrences in evolutionary history, the C_4_ pathway has molecularly evolved from modification to pre-existing enzymes and regulatory networks within C_3_ ancestors, rather than the evolution of completely new genes and traits (Monson, 1999; Gowik and Westhoff, 2011). Regarding leaf anatomy, it was shown that also in C_3_ plants, cells around the veins have a slightly different cellular morphology compared with the rest of the mesophyll cells, and so are termed pre-bundle sheath cells (Kinsman and Pyke, 1998). Pre-bundle sheath cell chloroplasts are smaller and occur at a lower density in the cell, and are often positioned on the cell wall distal to the vasculature (Kinsman and Pyke, 1998). Also on the physiological level, it was shown that in C_3_ plants, similar biochemical attributes to those needed in C_4_ photosynthesis are already present, being associated with photosynthesis around the vascular system of stems and petioles (Hibberd and Quick, 2002). The exact physiological role of BS cells in C_3_ plants is not fully understood; analysis of transcript residency on ribosomes in the *Arabidopsis thaliana* BS has revealed a role for the BS cells in sulfur and glucosinolate metabolism (Leegood, 2008; Aubry *et al.*, 2014).

Quantitative modelling has shown that C_4_ evolution proceeded stepwise and that each evolutionary step has contributed to an increase in the general fitness of the plant (Heckmann *et al.*, 2013). Extensive analysis of the *Flaveria* family, containing both C_3_ and C_4_ species within the family, as well as C_3_–C_4_ intermediates in several stages of evolution, has contributed enormously to the current knowledge on the evolutionary progression of C_3_ photosynthesis towards C_4_ photosynthesis (McKown *et al.*, 2005; Gowik *et al.*, 2011; Mallmann *et al.*, 2014). The first steps believed to activate the C_4_ photosynthetic programme in C_3_ plants are the inflating of the pre-bundle sheath cells accompanied by an increase in the numbers of chloroplasts and mitochondria, and the decrease of glycine decarboxylase (GDC) activity in the mesophyll cells (Bauwe, 2011; Sage *et al.*, 2012). GDC decarboxylates glycine that is formed in photorespiration. During the decarboxylation of glycine, CO_2_ is released as a by-product. The increase in the numbers of mitochondria and chloroplasts in the BS cells and the decrease of GDC activity in the mesophyll force the glycine formed by photorespiration in the mesophyll to migrate to the BS for decarboxylation, with the released CO_2_ accumulating and increasing Rubisco efficiency (Mallmann *et al.*, 2014; Sage *et al.*, 2014). This process is known as the photorespiratory CO_2_ pump (Bauwe, 2011)

The promoter of the bundle-specific expressed gene encoding the P-subunit of glycine decarboxylase (GLDPA) from the C_4_ species *Flaveria trinervia* maintains its bundle-specific expression when expressed in *A. thaliana* (Engelmann *et al.*, 2008). Conversely, the promoter of the gene encoding the sulfate transporter SULTR2;2 from *A. thaliana* acts in a bundle-specific manner when expressed in the C_4_ species *Flaveria bidentis* (Kirschner *et al.*, 2018). These two studies suggest a common transcriptional regulatory mechanism around the BS cells in C_3_ and C_4_ species. Recently, it was proven to be possible to create a reporter line in *A. thaliana* with chloroplast-targeted green fluorescent protein (GFP) under the control of the *GLDPA*_*Ft*_ bundle-specific promoter and to use this reporter line (the p*GLDPA*_*Ft*_*::RbcS.TP*-s*GFP* reference line) to obtain BS anatomy mutants (Döring *et al.*, 2019). One such mutational approach is activation tagging, in which a particular promoter is randomly inserted in a reference genome, resulting in alteration of the transcription pattern of genes in the proximity of the landing point of the inserted promoter (Tani *et al.*, 2004). In this study, the bundle-specific promoter of the gene encoding the T-subunit of GDC from the C_4_ species *F. trinervia*, proven to also be bundle specific in Arabidopsis (Emmerling, 2018), was used for tissue-specific activation tagging in the *A. thaliana* p*GLDPA*_*Ft*_*::RbcS.TP*-s*GFP* reference line.

By using activation tagging, this study identifies *NAC052*, a member of the NAC transcription factor gene family involved in post-transcriptional gene regulation (Butel *et al.*, 2017), as a novel genetic regulator of leaf morphology and bundle sheath anatomy in Arabidopsis.

## Materials and methods

### The *Arabidopsis thaliana* bundle sheath reporter line

An *A. thaliana* BS reporter line was used that contained a construct harbouring the promoter of the *GLDPA* gene (NCBI accession no. Z99767), a chloroplast transit peptide (TP) of the Arabidopsis RbcS gene, and a synthetic GFP (sGFP), termed the p*GLDPA*_*Ft*_*::RbcS.TP*-s*GFP* reporter line (Döring *et al.*, 2019).

### Activation tagging

The promoter sequence of the *GLDT* gene from *F. trinervia* (NCBI accession no. Z99769) was donated by J. Emmerling (Emmerling, 2018), amplified using PCR with restriction sites (*Pme*I and *Sac*I) added to the PCR primers, and inserted in the pMDC123 vector (Curtis and Grossniklaus, 2003), as close to the T-DNA left border as a unique restriction site was found to use for inserting the *GLDT* promoter (the restriction site *Pme*I was chosen). This *pMDC123-GLDT* vector was transformed in the Arabidopsis p*GLDPA*_*Ft*_*::RbcS.TP*-s*GFP* reporter line.

### Cloning of p*GLDT*_*Ft*_*::NAC052* and p*GLDT*_*Ft*_*::5'truncatedNAC052* constructs

The promoter of the *GLDT* gene from *F. trinervia* was inserted in the pAUL1 vector (Lyska *et al.*, 2013). The coding sequence of *NAC052* was isolated from cDNA from the Columbia-0 accession of *A. thaliana* using the primers listed in Supplementary Table S1 at *JXB* online.

To introduce the (truncated) *NAC052* coding sequence (CDS) into the Gateway entry vector pDONR221, the BP Clonase reaction (Gateway ‘BP Clonase II’ enzyme mix, ThermoFisher Scientific) was carried out as described by the manufacturer. The resulting pENTRY221-(truncated)NAC052 was subsequently used for the LR Clonase reaction (Gateway ‘LR Clonase II’ enzyme mix, ThermoFisher Scientific) to transfer the (truncated )*NAC052* CDS into p*AUL1-GLDT*_*Ft*_ (p*AUL1-GLDT*_*Ft*_*::NAC052* and p*AUL1-GLDT*_*Ft*_*::5'truncatedNAC052*).

### CRISPR/Cas

The target site for Cas9 was chosen in the first exon of *NAC052*, using the primers shown in Supplementary Table S1.

The primers were annealed to produce a single-guide RNA (sgRNA). The product was ligated in the *Bbs*I-digested sgRNA subcloning vector pFH6 (GenBank accession no. KY080689; Hahn *et al.*, 2017). The sgRNA cassette including the 20 bp target site was amplified from pFH6 (Table S1) and integrated into the *Kpn*I/*Hin*dIII-digested pUB-Cas9 vector (GenBank accession no. KY080691 (Hahn *et al.*, 2017)) via Gibson Assembly.

### Transformation of *A. thaliana*

The construct for transformation was inserted into the *Agrobacterium tumefaciens* strain AGL1 (Lazo *et al.*, 1991). After confirming the vector sequence (LGC Genomics, Berlin, Germany), the *A. tumefaciens* bacteria were put on the plant following the floral dip protocol (Clough and Bent, 1998), as modified by Logemann *et al.* (2006).

Depending on the nature of the construct, plants were selected on half-strength Murashige and Skoog (1/2 MS) plates containing 0.6% agar, 1% sucrose, and 50 μg ml^–1^ kanamycin or on soil (Floraton 1, Floragard, Oldenburg, Germany) watered with 80 mg l^–1^ glufosinate-ammonium (Bayer Agrar, Germany) and 0.1% Tween-20. Positive transformants were screened under the microscope for GFP signal.

### Light microscopy

The first leaf of ~2-week-old plants was analysed with a fluorescence microscope (Axio Imager M2m Zeiss, Oberkochen, Germany). The total GFP signal per leaf was quantified and normalized to leaf area with ImageJ (Version 2.0.0-rc-44/1.50e).

### Leaf sectioning for internal leaf anatomy

Internal leaf anatomy was assessed on sections sampled from the middle of the second leaf pair (one leaf per plant: three plants per line). The sections were prepared for light microscopy as described by Khoshravesh *et al.* (2017).

### Thermal asymmetric interlaced PCR (TAIL-PCR)

To determine the T-DNA insertion site after activation tagging, TAIL-PCR was performed as described by Singer and Burke (2003).

### Quantitative reverse–transcription PCR (qRT–PCR)

RNA was extracted from full-grown rosette leaves (five biological replicates) from non-flowering plants grown in soil (Floraton 1, Floragard) for 28 d in a climate-controlled growth chamber (16 h, 22 °C, 110 μmol m^−2^ s^−1^ light; 8 h, 20 °C dark); the RNA was extracted according to Onate-Sánchez and Vicente-Carbajosa (2008). After normalization of RNA concentrations, cDNA was synthesized using the Qiagen Quantitect Reverse Transcription Kit (Qiagen, Hilden, Germany). qRT–PCR was performed with three technical replicates for each biological replicate using the SYBR-green mastermix from KAPA SYBR FAST (KAPA Biosystems, Roche Sequencing and Life Science). Actin (At3g18780) was used as the reference gene for normalization of samples. The primers used are given in Supplementary Table S1.

### Reverse transcription–PCR (RT–PCR)

The presence of the predicted 5'truncated transcript variant was detected with agarose gel electrophoresis after RT–PCR using the primers listed in Supplementary Table S1.

### RNA sequencing

RNA was extracted from plants (three biological replicates) grown in the same conditions as the plants used for qRT–PCR, using the Qiagen RNeasy Plant Mini Kit including on-column DNA digestion. Total RNA samples were quantified (Qubit RNA HS Assay, Thermo Fisher Scientific) and quality was measured by capillary electrophoresis using the Fragment Analyzer and the ‘Total RNA Standard Sensitivity Assay’ (Agilent Technologies, Inc., Santa Clara, CA, USA). The library preparation was performed according to the manufacturer’s protocol using the Illumina^®^ ‘TruSeq Stranded mRNA Library Prep Kit’. Briefly, 200 ng of total RNA was used for mRNA capturing, fragmentation, the synthesis of cDNA, adaptor ligation, and library amplification. Bead-purified libraries were normalized and finally sequenced on the HiSeq 3000/4000 system (Illumina Inc., San Diego, CA, USA) with a read setup of 1×150 bp. The bcl2fastq tool was used to convert the bcl files to fastq files as well for adaptor trimming and demultiplexing.

Data analyses on fastq files were conducted with CLC Genomics Workbench (version 10.1.1, QIAGEN, Venlo, The Netherlands). The reads of all probes were adaptor trimmed (Illumina TruSeq) and quality trimmed (using the default parameters: bases below Q13 were trimmed from the end of the reads, ambiguous nucleotides maximal 2). Mapping was done against the *A. thaliana* (TAIR10) (25 May 2017) genome sequence as described by Mortazavi *et al.* (2008). A principal component analysis (PCA) was performed to analyse the correlation between the biological replicates. The DESeq2 R package was used to determine the differentially expressed genes (Love *et al.*, 2014). The Gene Ontology (GO) Analysis Toolkit and Database for Agricultural Community (AgriGO) was use for GO enrichment analysis (http://systemsbiology.cau.edu.cn/agriGOv2/). The RNA sequencing data set has been deposited at NCBI with accession number GSE139503, and can be accessed through https://www.ncbi.nlm.nih.gov/geo/query/acc.cgi?acc=GSE139503.

## Results

### Activation tagging

**Fig. 1.**
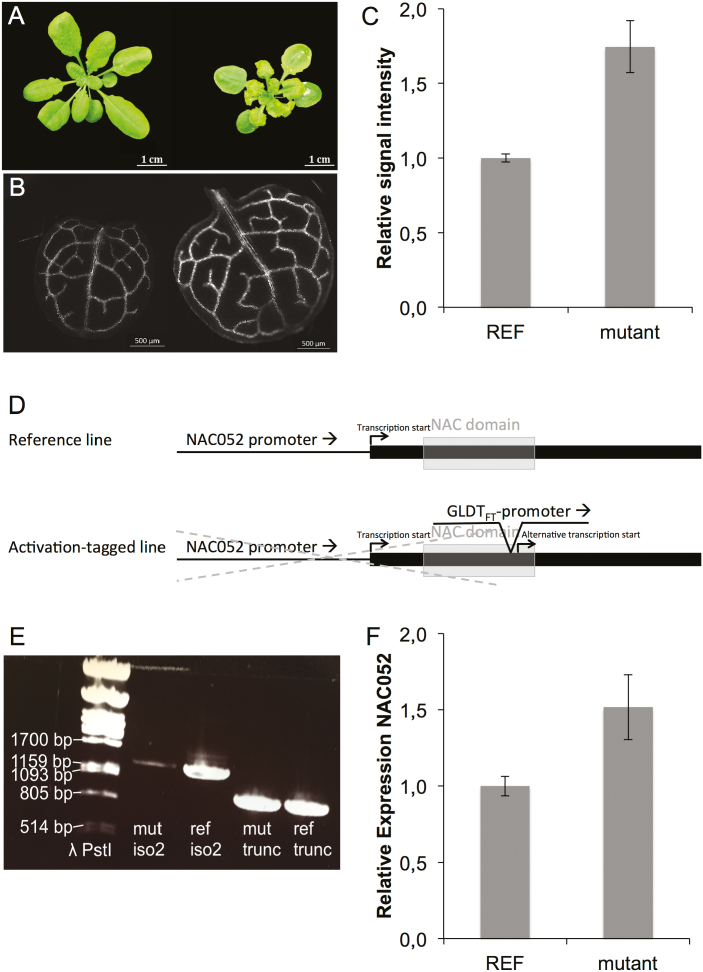
C_4_ promoter-induced expression of NAC052 increases signal intensity of reporter gene expression in the leaf bundle and changes leaf morphology of C_3_*Arabidopsis thaliana.* (A) Leaf morphology of 28-day-old *A. thaliana* reference line (Col-0 transformed with GFP under control of the *Flaveria trinervia*-derived *GLDPA* promoter) and the mutant line (reference line transformed with activation tagging construct with the *F. trinervia*-derived GLDT promoter). (B) GFP signal overview of first leaf of 14-day-old reference line and mutant line. (C) Quantification of GFP signal of first leaf of 14-day-old reference line and mutant line, *n*=5. (D) Overview of the genomic landing point of the *F. trinervia*-derived GLDT promoter in the activation-tagged line. An alternative start codon in-frame with the other two ATGs was discovered 32 bp downstream of the genomic landing point of the GLDT promoter. The grey dotted line represents disfunction of the endogenous NAC052 promoter in the activation-tagged line. (E) Presence/absence (RT–PCR) of the wild-type transcript nr2 and of the alternative 5'truncated transcript. (F) Quantification (qRT–PCR) of the *NAC052* transcripts in the reference and mutant line.

A *GLDT*_*Ft*_ promoter activation-tagged line was identified with an increased signal intensity of the GFP reporter gene expressed in the leaf bundle, and a small rosette area with wrinkled leaves and chlorotic leaf edges (Fig. 1A–C). Genomic analysis revealed that the *F. trinervia-*derived *GLDT* promoter (the activation tag) had inserted in the coding region of the gene encoding the transcription factor NAC052 (At3g10490), a transcriptional repressor functioning in H3K4 demethylation (Ning *et al.*, 2015; Zhang *et al.*, 2015). This gene is also known as *SUPPRESSOR OF GENE SILENCING 1* (*SGS1*), named as such because its downstream effect is on genes that are crucial for post-transcriptional gene silencing (Butel *et al.*, 2017). Four splice variants are known for *NAC052* from published RNA sequencing experiments (Cheng *et al.*, 2017; Supplementary Fig. S1). The different splice variants are expressed in different parts of the plant; only transcripts nr2 and nr4 were measured in the leaf, of which transcript nr2 was most prominent (Cheng *et al.*, 2017). Our *GLDT*_*Ft*_ promoter had inserted in the end of the NAC domain; sequence analysis revealed an alternative ATG start site 30 bp downstream of the *GLDT*_*Ft*_ promoter insertion, in-frame with the coding sequence (Fig. 1D). We hypothesized the *GLDT*_*Ft*_ insertion led to production of an aberrant, 5'truncated transcript variant of *NAC052*. We could not detect full-length transcript nr2 in our activation-tagged mutant line (Fig. 1E). However, we could detect an increase in RNA quantity of the later exons (Fig. 1F).

### NAC052 is involved in leaf development

**Fig. 2.**
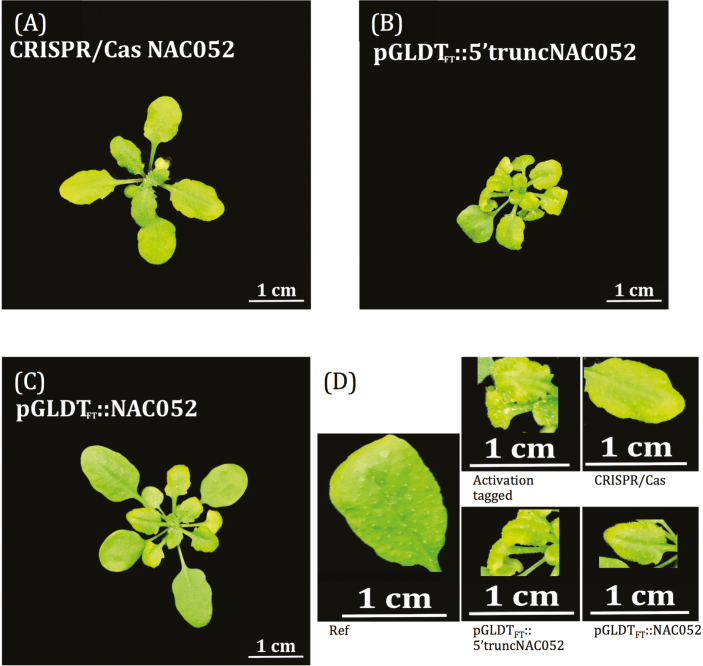
Leaf morphology of 28-day-old rosettes of *Arabidopsis thaliana.* (A) CRISPR/Cas line (T_3_), (B) p*GLDT*_*Ft*_*::5'truncatedNAC052* line (T_1_), and (C) p*GLDT*_*Ft*_*::NAC052* line (T_1_). (D) Close-ups of the leaves from the reference line, the activation-tagged line, the CRISPR/Cas line, the *pGLDT*_*FT*_*::5'truncatedNAC052* line, and the p*GLDT*_*Ft*_*::NAC052* line to highlight leaf morphological traits.

**Fig. 3.**
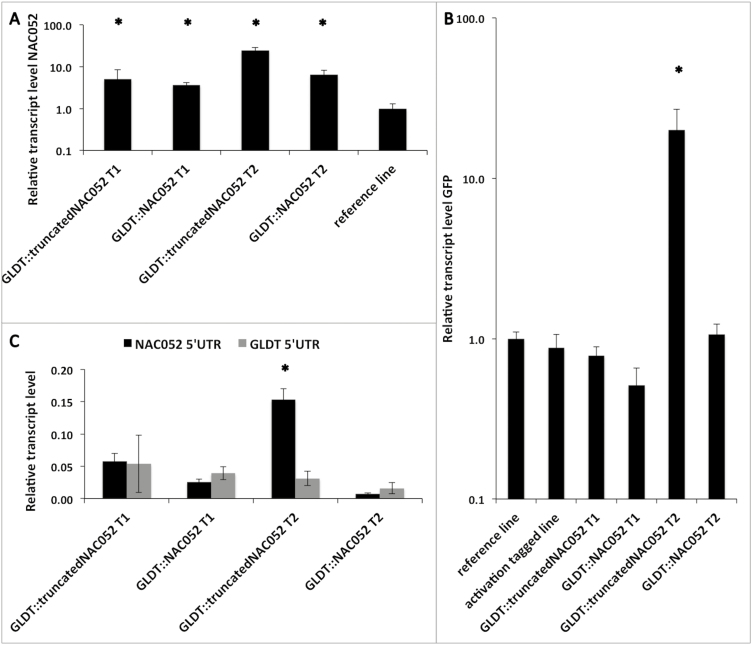
Quantification (qRT–PCR) of transcripts in transformed lines of *Arabidopsis thaliana.* (A) *NAC052* (normalized to reference line), (B) *GFP* transgene (normalized to reference line), (C) *NAC052* endogene (*NAC052* 5'UTR) versus *NAC052* transgene (*GLDT* 5'UTR); quantification relative to reference gene used for qRT–PCR

We tried to reconstruct the mutation event of our activation-tagged line by expressing p*GLDT*_*Ft*_*::5'truncatedNAC052* in the p*GLDPA*_*Ft*_*::RbcS.TP*-s*GFP* reference line and could confirm enhanced GFP signal intensity in 1/10 T_1_ plants (70 T_1_ plants were analysed). These T_1_ plants were smaller than the untransformed reference line and had chlorotic and wrinkled leaf edges, similar to the original activation-tagged line (Fig. 2). The level of endogenous *NAC052* transcripts was increased in the T_1_ plants with enhanced GFP signal intensity (Fig. 3A), suggesting that the expression of the 5'truncated *NAC052* gene influences transcript levels of the endogenous *NAC052*. However, in the T_2_ plants, the GFP signal was reduced compared with the untransformed reference line and the leaf morphology phenotype was lost in all plants. This suggests that the p*GLDT*_*Ft*_*::5'truncatedNAC052* construct somehow was silenced. To confirm this, we measured the transcript level of the *GFP* gene itself. The p*GLDT*_*Ft*_*::5'truncatedNAC052* transgene increased the transcript level of the *GFP* gene only in the T_2_ generation, but not in T_1_ (Fig. 3B). However, the increased transcript level of the *GFP* gene did not lead to an increase in the GFP signal observed in the T_2_ plants. The activation-tagged line, which also accumulates a *5'truncated NAC052* transcript variant, but has no endogenous *NAC052* function (Fig. 1), showed no effect on the transcript levels of the *GFP* transgene (Fig. 3B). However, the activation-tagged line exhibited an increased GFP signal (Fig. 1). This suggests that the endogenous native copy of *NAC052* present in the genomic background of the p*GLDT*_*Ft*_*::5'truncatedNAC052* line somehow post-transcriptionally silences the *GFP* gene. This inference is strengthened by the observation of increased transcript levels of the endogenous *NAC052* in the T_2_ generation of the p*GLDT*_*Ft*_*::5'truncatedNAC052* line (Fig. 3C).

For further confirmation of NAC052 involvement in leaf development, we expressed the entire reading frame of *NAC052* transcript nr2 under the control of the *GLDT*_*Ft*_ promoter in the p*GLDPA*_*Ft*_*::RbcS.TP*-s*GFP* reference line, creating a p*GLDT*_*Ft*_*::NAC052* transgenic line. We could confirm enhanced GFP signal intensity in 4/10 T_1_ plants (40 T_1_ plants were analysed). In addition, these T_1_ plants were smaller than the untransformed reference line and had chlorotic leaf edges (Fig. 2). In contrast to the activation-tagged line and to the T_1_ plants from the p*GLDT*_*Ft*_*::5'truncatedNAC052* line, the T_1_ plants from the p*GLDT*_*Ft*_*::NAC052* line did not show wrinkled leaf edges. Again, the phenotype was completely lost in the T_2_ generation. The transcript levels of the *GFP* gene were unaltered in both the T_1_ and T_2_ generations of the p*GLDT*_*Ft*_*::NAC052* line (Fig. 3B). Unlike in the p*GLDT*_*Ft*_*::5'truncatedNAC052* line, the transcripts levels of endogenous *NAC052* were not increased in the T_2_ generation (Fig. 3C).

For even further confirmation of the involvement of NAC052 in leaf development, we mutated the endogenous *NAC052* with clustered regularly interspaced short palindromic repeats (CRISPR)/CRISPR-associated protein (Cas). In this CRISPR/Cas mutant, a thymine nucleotide was inserted in the first exon of wild-type transcript nr2 of *NAC052,* leading to a frameshift (Supplementary Fig. S2). Similar to the activation-tagged mutant and to the T_1_ plants of the p*GLDT*_*Ft*_*::5'truncatedNAC052* line, the CRISPR/Cas mutant was small and had chlorotic leaf edges (Fig. 2), but it did not show wrinkled leaf edges. However, in contrast to the activation-tagged mutant, the GFP signal intensity was decreased in the CRISPR/Cas mutant as compared with the reference line. Thus, the dysfunctional *NAC052* transcript variant induced by CRISPR/Cas and the dysfunctional 5'truncated *NAC052* transcript variant appear to function differently.

**Fig. 4.**
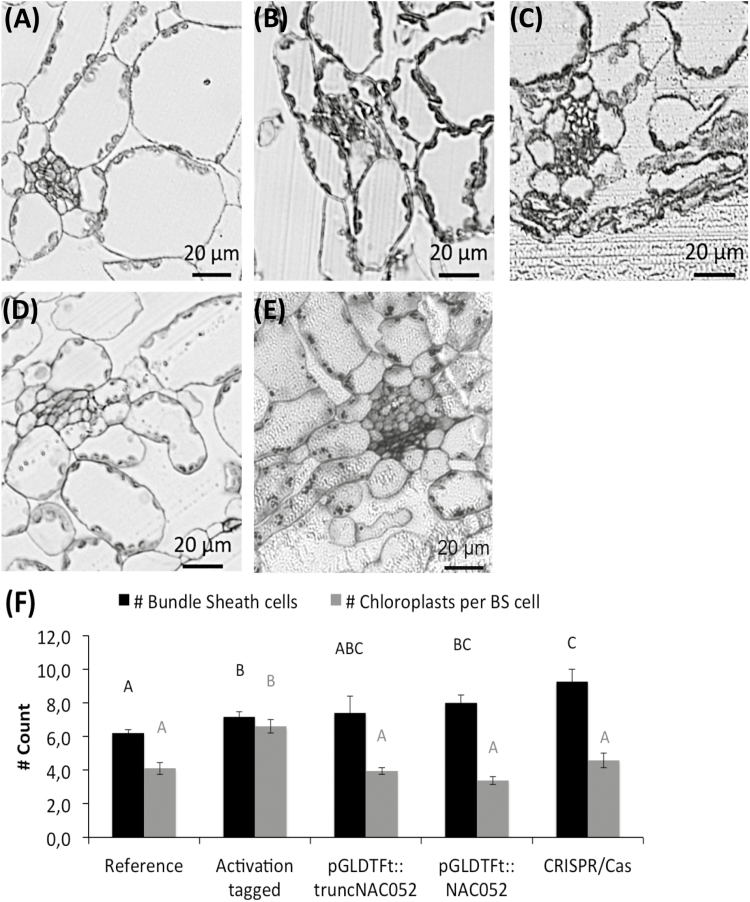
Light micrographs illustrating transverse cross-sections of a third-order vein. (A) Reference line, (B) activation-tagged line, (C) p*GLDT*_*Ft*_*::5'truncatedNAC052* line, (D) p*GLDT*_*Ft*_*::NAC052* line, (E) CRISPR/Cas line. (F) Quantification of the number of BS cells and the number of chloroplasts per BS cell. Letters indicate statistically significant differences as determined by ANOVA (*n*=5; *P*<0.05)

In order to fully characterize the function of NAC052 in leaf development and BS anatomy, multiple measurements were done on the transgenic plants that were created. To provide an overview, the results of those measurements are summarized in Table 1. To see the effect of the bundle-specific expression of *5'truncated NAC052* on internal leaf anatomy, transverse cross-sections of leaves of the activation-tagged, the p*GLDT*_*Ft*_*::5'truncatedNAC052*, the p*GLDT*_*Ft*_*::NAC052*, and the CRISPR/Cas lines were compared with cross-sections of the reference line. The activation-tagged line showed enhanced numbers of cells in the BS tissue compared with the reference line, and those cells contained a higher number of chloroplasts (Fig. 4). No such increases were observed in the mesophyll cells. Similar to the activation-tagged line, the p*GLDT*_*Ft*_*::5'truncatedNAC052*, the p*GLDT*_*Ft*_*::NAC052* line (both in the T_2_ generation), and the CRISPR/Cas line showed increased number of cells in the BS tissue (Fig. 4). However, in contrast to the activation-tagged line, the numbers of chloroplasts in the BS cells of the p*GLDT*_*Ft*_*::5'truncatedNAC052* and the p*GLDT*_*Ft*_*::NAC052* lines were not increased compared wirtt the reference line (Fig. 4).

**Table 1.** Overview of the characteristics regarding NAC052 function in the lines constructed for confirmation of NAC052 involvement in bundle sheath ontogeny

Line	GFP signal compared with reference line	NAC052 read count (*n*=3)	NAC052 endogenous function?	Truncated NAC052 function?	No. of genes with differential read count compared with reference line	No. of BS cells (*n*=5)	BS anatomy compared with reference line
Reference	–	657±32	Yes	No	–	6.2±0.2	–
Activation tagged	Increased	2106±337	No	Yes	69	7.2±0.3	More BS cells, more chloroplasts
CRISPR/Cas	Reduced	849±31	No	No	1028	NM	NM
p*GLDT*_*Ft*_*::5'truncated NAC052*	Reduced	1743±425	Yes	Yes	65	7.4±1.0	More BS cells
p*GLDT*_*Ft*_*::NAC052*	Same	6229±2910	Yes	No	1310	8.0±0.4	More BS cells

NM=not measured.

### Downstream genes affected by NAC052

**Fig. 5.**
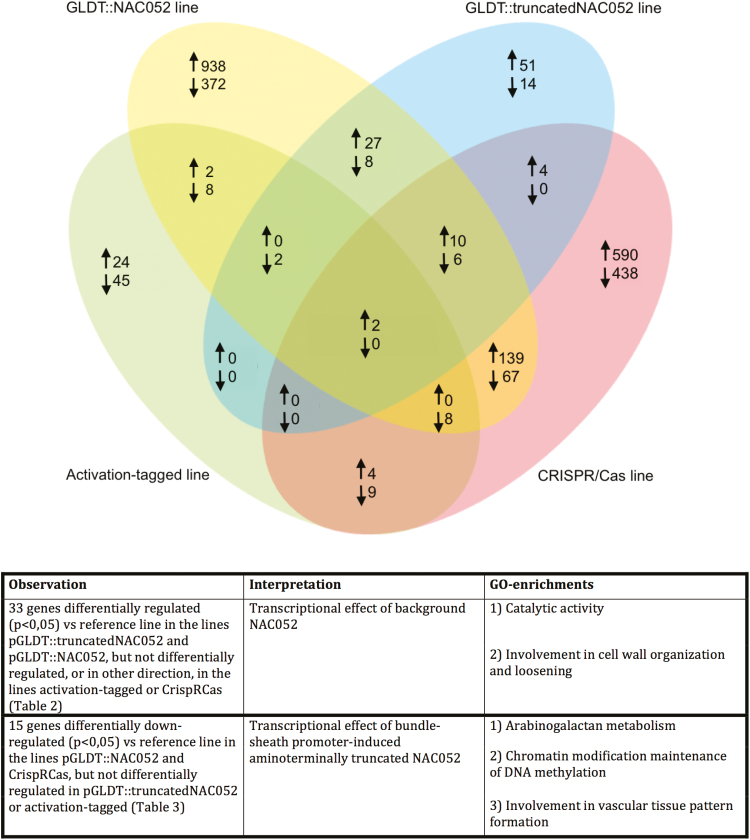
Number of genes with a differential transcript count compared with the p*GLDPA*_*Ft*_*::RbcS.TP*-s*GFP* reference line. Venn diagrams displaying the number of significantly (*P*=0.05) differentially (>2.0-fold up- or down-regulated) expressed genes when comparing the p*GLDT*_*Ft*_*::NAC052*, p*GLDT*_*Ft*_*::5'truncatedNAC052*, activation-tagged, or CRISPR/Cas transformed line with the reference line. The panel gives an overview of the GO enrichments.

To get a clearer idea of which downstream genes and what biological processes are affected by targeted misexpression of NAC052, we performed mRNA sequencing on the activation-tagged line, the p*GLDT*_*Ft*_*::5'truncatedNAC052* transgenic line, the p*GLDT*_*Ft*_*::NAC052* transgenic line, and the CRISPR/Cas mutated line. Figure 5 shows the genes with differential transcript counts compared with the p*GLDPA*_*Ft*_*::RbcS.TP*-s*GFP* reference line (fold change >2 or <0.5; *P*<0.05); the correlation between the biological replicates is shown in Supplementary Fig. S3; mapping of the reads to the four different splice variants of NAC052 as well as the expression of the splice variants is shown in Supplementary Figs S4 and S5. The activation-tagged line and the p*GLDT*_*Ft*_*::5'truncatedNAC052* line showed a low number of differentially counted transcripts compared with the reference line, whereas the p*GLDT*_*Ft*_*::NAC052* line and the CRISPR/Cas mutated line showed a relatively high number of differentially counted transcripts compared with the reference line (Fig. 5). No significant GO enrichments were found within the descriptions of the 69 genes that are differentially regulated in the activation-tagged line when compared with the reference line, nor were any GO enrichments found among the 65 genes that are differentially regulated in the p*GLDT*_*Ft*_*::5'truncatedNAC052* line when compared with the reference line. No genes respond similarly in the activation-tagged line as in the p*GLDT*_*Ft*_*::5'truncated NAC052* line when compared with the reference line (Fig. 5).

Based on the introduced mutations, both in the activation-tagged line and in the CRISPR/Cas line, the endogenous full-size NAC052 protein should be absent, while the p*GLDT*_*Ft*_*::5'truncatedNAC052* and the p*GLDT*_*Ft*_*::NAC052* lines should still accumulate endogenous NAC052 protein in the background. We compared descriptions of genes that are similarly responsive in the p*GLDT*_*Ft*_*::5'truncatedNAC052* and the p*GLDT*_*Ft*_*::NAC052* line, as well as similarly responsive in the activation-tagged line and the CRISPR/Cas line, but differently responsive between p*GLDT*_*Ft*_*::5'truncatedNAC052*/p*GLDT*_*Ft*_*::NAC052* and activation-tagged/CRISPR/Cas. In such a way, we could investigate the transcriptional effect of endogenous NAC052 protein expression in the background. Thirty-three genes respond in such a way (Table 2). These genes are enriched for the GO terms ‘catalytic activity’ and ‘involvement in cell wall organization and loosening’. The genes involved in catalytic activity all encode enzymes, for example a temperature-sensitive plastidic fatty acid desaturase, a cysteine-rich receptor-like protein kinase, and a calcium-dependent phosphotriesterase superfamily protein. Among the genes involved in cell wall organization are, for example, a xyloglucan endotransglucosylase/hydrolase, an aspartyl protease, and an expansin-like protein.

**Table 2.** Genes that are similarly responsive in the *GLDT*_*Ft*_*::5'truncatedNAC052* and the *GLDT*_*Ft*_*:NAC052* lines, but differently responsive in the activation-tagged line and the CRISPR/Cas line

	Gene ID	Gene description	Abbreviated gene name	Activation tagged	CRISPR/Cas	*GLDT:: truncatedNAC052*	*GLDT:: NAC052*
1	AT1G01060	LHY encodes a myb-related putative transcription factor (TF) involved in circadian rhythm along with another myb TF CCA1.	LHY	2.06	2.37	0.5<FC<2.0	0.5<FC<2.0
2	AT1G16730	UNKNOWN PROTEIN 6, expressed during flowering stage, petal differentiation, and expansion stage; expressed in guard cell.	UP6	2.10	2.44	0.5<FC <2.0	0.5<FC<2.0
3	AT4G40020	Myosin heavy chain-related protein; involved in reciprocal meiotic recombination.		3.34	2.37	0.5<FC<2.0	0.5<FC<2.0
4	AT5G05580	Encodes a temperature-sensitive plastidic fatty acid desaturase. Located in chloroplast, expressed in guard cell.	FAD8	0.5<FC<2.0	0.5<FC<2.0	0.47	0.42
5	AT5G67370	CONSERVED IN THE GREEN LINEAGE; involved in response to iron ion starvation; located in chloroplast, integral component of membrane.	CGLD27	0.5<FC<2.0	0.5<FC<2.0	0.47	0.32
6	AT1G72416	Chaperone DnaJ-domain superfamily protein; located in cytoplasm, integral component of membrane; expressed in guard cell.		0.5< FC <2.0	0.5< FC <2.0	0.44	0.45
7	AT2G34510*	Choice-of-anchor C domain protein, putative; expressed in cauline leaf, collective leaf structure, cotyledon.		0.5<FC<2.0	0.5<FC<2.0	0.45	0.33
8	AT4G11460*	Encodes a cysteine-rich receptor-like protein kinase, involved in defence response to bacteria, protein phosphorylation.	CRK30	0.5<FC<2.0	0.5<FC<2.0	0.42	0.36
9	AT5G01015*	Transmembrane protein.		0.5<FC<2.0	0.5<FC<2.0	0.35	0.26
10	**AT1G74010**	Calcium-dependent phosphotriesterase superfamily protein; located in cytosol, endoplasmic reticulum, extracellular region, membrane, plant-type cell wall.		0.5<FC<2.0	0.5<FC<2.0	2.13	5.03
11	AT2G22880	VQ motif-containing protein; involved in response to UV-B; located in nucleus.	VQ12	0.5<FC<2.0	0.5<FC<2.0	2.52	9.60
12	**AT2G39350**	Belongs to a clade of five *Arabidopsis thaliana* ABCG half-transporters that are required for synthesis of an effective suberin barrier in roots and seed coats (ABCG2, ABCG6, and ABCG20) and for synthesis of an intact pollen wall (ABCG1 and ABCG16).	ABCG1	0.5<FC<2.0	0.5<FC<2.0	2.71	7.97
13	AT2G39980	HXXXD-type acyl-transferase family protein; involved in response to karrikin; has transferase activity.		0.5<FC<2.0	0.5< FC <2.0	2.17	3.71
14	AT4G06410	Natural antisense transcript overlaps with AT4G16670.		0.5<FC<2.0	0.5<FC<2.0	2.28	5.53
15	AT4G15550	IAGLU, INDOLE-3-ACETATE BETA-d-GLUCOSYLTRANSFERASE.	IAGLU	0.5< FC <2.0	0.5< FC <2.0	2.58	2.51
16	AT4G25810*	Xyloglucan endotransglycosylase-related protein (XTR6); involved in cell wall biogenesis, cell wall organization, xyloglucan metabolic process; located in Golgi apparatus, apoplast, cell wall, extracellular region.	XTR6	0.5<FC<2.0	0.5<FC<2.0	2.00	5.71
17	AT4G35770	Senescence-associated gene that is strongly induced by phosphate starvation. Transcripts are differentially regulated at the level of mRNA stability at different times of day.	SEN1	0.5<FC<2.0	0.5<FC<2.0	2.09	2.66
18	AT5G13330*	Encodes a member of the ERF (ethylene response factor) subfamily B-4 of ERF/AP2 transcription factor family.	Rap2.6L	0.5<FC<2.0	0.5<FC<2.0	2.12	4.55
19	AT1G75450	This gene used to be called AtCKX6. It encodes a protein whose sequence is similar to cytokinin oxidase/dehydrogenase.	CKX5	0.50	0.30	2.00	2.65
20	AT2G19800	Encodes a myo-inositol oxygenase family gene, involved in l-ascorbic acid biosynthetic process, inositol catabolic process, oxidation–reduction process, syncytium formation.	MIOX2	0.36	0.50	2.00	2.30
21	AT3G50970	Belongs to the dehydrin protein family, involved in cold acclimation, defence response to fungus, response to abscisic acid, response to cold, response to water, response to water deprivation; the mRNA is cell-to-cell mobile.	LTI30	0.28	0.46	2.00	3.23
22	AT4G20970	Basic helix–loop–helix (bHLH) DNA-binding superfamily protein; has DNA-binding transcription factor activity.		0.39	0.42	2.00	2.00
23	AT4G21650*	Subtilase family protein; involved in proteolysis.	SBT3.13	0.36	0.48	2.00	2.00
24	AT5G07000	Encodes a member of the sulfotransferase family of proteins. It may be able to act on structurally related jasmonates.	ST2B	0.41	0.23	0.5<FC<2.0	0.5<FC<2.0
25	**AT5G55250**	Encodes an enzyme that specifically converts IAA to its methyl ester form MelIAA, involved in auxin homeostasis, methylation, polarity specification of adaxial/abaxial axis.	IAMT1	0.47	0.34	0.5<FC<2.0	0.5<FC<2.0
26	AT1G04220	Encodes KCS2, a member of the 3-ketoacyl-CoA synthase family involved in the biosynthesis of VLCFA (very long chain fatty acids).	KCS2	0.5<FC<2.0	0.5<FC<2.0	2.16	3.01
27	AT1G64660	Encodes a functional methionine gamma-lyase, a cytosolic enzyme catalyses the degradation of methionine into methanethiol, alpha-ketobutyrate, and ammonia.	MGL	0.5<FC<2.0	0.5<FC<2.0	2.05	3.26
28	AT2G23170	Encodes an IAA-amido synthase that conjugates Asp and other amino acids to auxin *in vitro*.	GH3.3	0.5<FC<2.0	0.5<FC<2.0	2.57	4.20
29	AT3G54400*	Eukaryotic aspartyl protease family protein; involved in protein catabolic process, proteolysis; located in apoplast, cell wall, chloroplast, extracellular region, plant-type cell wall.		0.5<FC<2.0	0.5<FC<2.0	2.12	4.24
30	**AT3G55500**	Expansin-like protein, involved in plant-type cell wall loosening, plant-type cell wall modification involved in multidimensional cell growth, plant-type cell wall organization, syncytium formation, unidimensional cell growth.	ExPA16	0.5<FC<2.0	0.5<FC<2.0	2.01	2.54
31	AT3G62090	PHYTOCHROME-INTERACTING FACTOR 6, encodes a novel Myc-related bHLH transcription factor, which physically associated with APRR1/TOC1 and is a member of PIF3 transcription factor family.	PIL2	0.5<FC<2.0	0.5<FC<2.0	2.06	2.27
32	AT5G06570*	Alpha/beta-Hydrolase superfamily protein; expressed during petal differentiation and expansion stage.		0.5<FC<2.0	0.5<FC<2.0	2.16	7.02
33	**AT2G32990**	Glycosyl hydrolase 9B8; involved in cell wall organization, cellulose catabolic process.	GH9B8	0.45	0.40	0.5<FC<2.0	0.5<FC<2.0

Fold changes are compared with the reference line. Gene IDs in bold are involved in cell wall organization; gene IDs that include ‘*’ overlap with up-regulated genes after *NAC050/052-RNAi* (Ning *et al.*, 2015); gene IDs underlined overlap with differentially expressed genes between total leaf and bundle sheath only (Aubry *et al.*, 2014); fold changes in blue are increased compared with the reference line (*P*=0.05); fold changes in red are decreased compared with the reference line (*P*=0.05).

Both the activation-tagged line and the p*GLDT*_*Ft*_*::5'truncated NAC052* line are exposed to altered NAC052 functionality due to an N-terminal truncation of the NAC052 protein. Because NAC052 is a transcriptional repressor (Zhang *et al.*, 2015), it is interesting to analyse descriptions of genes that are down-regulated (when compared with the reference line) in the p*GLDT*_*Ft*_*::NAC052* line as well as in the CRISPR/Cas line, but are not differentially expressed compared with the reference line in the activation-tagged line and the p*GL DT*_*Ft*_*::5'truncatedNAC052* line. Fifteen genes respond in such a way; three of those genes encode proteins functional in arabinogalactan metabolism, one is a chromatin modification maintainer of DNA methylation, and one is a carboxypeptidase involved in leaf vascular tissue pattern formation (Table 3).

**Table 3.** Genes that are similarly responsive in the *GLDT*_*Ft*_*:NAC052* line and CRISPR/Cas line, but not responsive in the activation-tagged line and in the *GLDT*_*Ft*_*::5'truncatedNAC052* line

	Gene ID	Gene description	Abbreviated gene name	Acti-vation tagged	CRISPR/Cas	*GLDT::5'truncatedNAC052*	*GLDT::NAC052*
1	AT1G02640*	Encodes a protein similar to a beta-xylosidase located in the extracellular matrix (AT5G49360). This is a member of glycosyl hydrolase family 3 and has six other closely related members.	BXL2	0.5<FC<2.0	0.36	0.5<FC<2.0	0.46
2	AT1G26820	Encodes ribonuclease RNS3. Involved in RNA catabolic process and ageing.	RNS3	0.5<FC<2.0	0.34	0.5<FC<2.0	0.50
3	**AT1G66040**	ORTH4, ORTHRUS 4, VARIANT IN METHYLATION 4, VIM4 Involved in chromatin organization, maintenance of DNA methylation, protein ubiquitination. Protein located in nucleus.	VIM4	0.5<FC<2.0	0.26	0.5<FC<2.0	0.44
4	AT1G70985	Hydroxyproline-rich glycoprotein family protein, protein located in anchored component of membrane. Expressed in seeds of first silique		0.5<FC<2.0	0.44	0.5<FC<2.0	0.50
5	AT1G78450*	SOUL haem-binding family protein, protein located in chloroplast, expressed in hypocotyl and siliques.		0.5<FC<2.0	0.41	0.5<FC<2.0	0.39
6	AT2G29300	NAD(P)-binding Rossmann-fold superfamily protein, protein location unknown, expressed in seeds of first silique.		0.5<FC<2.0	0.44	0.5<FC<2.0	0.48
7	AT3G13000	Ubiquinone biosynthesis protein, expressed in young leaf and young flower. Protein located in nucleus and vacuole.		0.5<FC<2.0	0.48	0.5<FC<2.0	0.50
8	**AT3G54720**	ALTERED MERISTEM PROGRAM 1, AMP1, encodes glutamate carboxypeptidase. Various alleles show increased cotyledon number and rate of leaf initiation, show transformation of leaves to cotyledons, altered flowering time and photomorphogenesis, and an increased level of cytokinin biosynthesis. Protein located in endoplasmic reticulum. Involved in leaf vascular tissue pattern formation.	AMP1	0.5<FC<2.0	0.47	0.5<FC<2.0	0.39
9	AT3G62070	Hypothetical protein expressed in guard cell.		0.5<FC<2.0	0.48	0.5<FC<2.0	0.39
10	AT4G03610	Metallo-hydrolase/oxidoreductase superfamily protein, protein located in nucleus, expressed in plant embryo.		0.5<FC<2.0	0.19	0.5<FC<2.0	0.45
11	AT4G20820	FAD-binding Berberine family protein, electron transfer activity, protein located in chloroplast, expressed in collective leaf structure, cotyledon, guard cell, hypocotyl, root, stem, vascular leaf.	ATBBE18	0.5<FC<2.0	0.29	0.5<FC<2.0	0.34
12	AT4G26288	Hypothetical protein, expressed in silique, protein located in nucleus		0.5<FC<2.0	0.47	0.5<FC<2.0	0.42
13	AT4G34790	SAUR-like auxin-responsive protein family, protein located in mitochondrion. Expressed in guard cell.	SAUR3	0.5<FC<2.0	0.27	0.5<FC<2.0	0.33
14	**AT4G40090**	Arabinogalactan protein 3, involved in multicellular organism development, protein located in anchored component of membrane, expressed in hypocotyl, plant egg cell, root, root hair cell, shoot apex, trichoblast.	AGP3	0.5<FC<2.0	0.49	0.5<FC<2.0	0.42
15	AT5G49360*	Encodes a bifunctional (beta)-d-xylosidase/(alpha)-l-arabinofuranosidase required for pectic arabinan modification. Located in the extracellular matrix. Gene is expressed specifically in tissues undergoing secondary wall thickening. This is a member of glycosyl hydrolase family 3 and has six other closely related members.	BXL1	0.5<FC<2.0	0.50	0.5<FC<2.0	0.46

Fold changes are compared with the reference line. Gene IDs in bold are involved in either chromatin organization, leaf vascular tissue pattern formation, or arabinogalactan functioning; gene IDs that include ‘*’ overlap with up-regulated genes after *NAC050/052-RNAi* (Ning *et al.*, 2015); gene IDs underlined overlap with differentially expressed genes between total leaf and bundle sheath only (Aubry *et al.*, 2014); fold changes in red are decreased compared with the reference line (*P*=0.05)

## Discussion

In this study, activation tagging led to expression of a 5'truncated form of the NAC052 transcription factor, in which the DNA-binding domain was partly deleted. In our leaf bundle-targeted GFP reporter line, this mutation led to changes in GFP fluorescence levels, as well as anatomical changes in the BS, in addition to changes in whole-plant and leaf morphology.

### NAC052 acts as a regulator of leaf development

*NAC052* belongs to the NAC [no apical meristem (NAM), Arabidopsis transcription activation factor (ATAF), Cup-shaped cotyledon (CUC)] family of transcription factors. NAC transcription factors typically posses the conserved N-terminal NAC domain (~150 amino acids), which contains the DNA-binding domain, and a diversified C-terminal transcription regulatory region (Puranik *et al.*, 2012). *NAC052* is a duplicated gene of *NAC050*, and they together bind DNA in the form of dimers and associate with the histone demethylase JMJ14, leading to histone H3K4 demethylation (Ning *et al.*, 2015; Zhang *et al.*, 2015). JMJ14 acts in several biological processes, including mobile RNA silencing, DNA methylation, abundance of endogenous transposon transcripts, and flowering time genes (Deleris *et al.*, 2010; Lu *et al.*, 2010; Searle *et al.*, 2010; Le Masson *et al.*, 2012). Of the two NAC transcription factors that associate with JMJ14, mutational effects in NAC052 are similar to *jmj14*-mutants, whereas mutations in NAC050 have only moderate effects (Zhang *et al.*, 2015). These mutational effects include suppression of post-transcriptional gene silencing (PTGS), leading to enhanced transcription levels of several endogenous targets of JMJ14 as well as reduced transcript levels of transgene loci (Searle *et al.*, 2010; Le Masson *et al.*, 2012). Within the RNA silencing process, JMJ14 was found to act downstream from the Argonaute effector complex to demethylate histone H3K4 at the RNA silencing target gene (Searle *et al.*, 2010). Increased H3K4me3 levels at endogenous loci correlate with increased transcription at the same loci (Zhang *et al.*, 2009). Whole-genome analysis of H3K4me3 levels and of RNA transcript levels in *jmj14* revealed 130 genes that were both hypermethylated and up-regulated in *jmj14*; none of these genes overlapped with genes found to be transcriptionally responsive to misexpressed NAC052 in this study (Tables 2, 3) (Ning *et al.*, 2015).

Besides identifying *jmj14*, forward genetic screening for mutants defective in PTGS has identified *suppressor of gene silencing 1* (*sgs1*), found to be impaired in the *NAC052* gene (Le Masson *et al.*, 2012; Butel *et al.*, 2017). Butel *et al.* (2017) showed that besides repressing transcription of endogenous genes that are involved in PTGS, the JMJ14–NAC052 module promotes transgene transcription by preventing DNA methylation, confirming that the JMJ14–NAC052 module has a dual effect (Searle *et al.*, 2010; Le Masson *et al.*, 2012). Whole-genome analysis of RNA transcript levels in an *NAC050/052-RNAi* plant identified 1470 genes with enhanced transcript levels (Ning *et al.*, 2015), of which eight genes overlapped with the genes found in this study that are differently responsive between p*GLDT*_*Ft*_*::5'truncatedNAC052*/p*GLDT*_*Ft*_*::NAC052* and activation-tagged/CRISPR/Cas (Table 2), and three genes overlapped with the genes that are differently responsive between p*GLDT*_*Ft*_*::NAC052/*CRISPR/Cas and p*GLDT*_*Ft*_*::5'truncated NAC052/*activation-tagged (Table 3).

In this study, an increased transcript level of an N-terminally truncated NAC052 in an activation-tagged line was observed. The N-terminally truncated NAC052 has 304 amino acids instead of the wild-type 452 amino acids, and it contains only part of the conserved NAC domain. This conserved NAC domain consists of five subdomains (A–E), and the crucial DNA-binding domain is situated within a 60 amino acid region located within subdomains D and E (Kikuchi *et al.*, 2000; Duval *et al.*, 2002). The N-terminally truncated NAC052 contains only subdomain E, but not A–D. Therefore, we hypothesize that the N-terminally truncated NAC052 has no functional DNA-binding domain and is an unable to associate with JMJ14, leading to a non-functional H3K4 demethylase.

When cloning the *F. trinervia*-derived *GLDT* promoter in front of the full-length CDS of *NAC052*, or in front of the 5'truncated *NAC052*, we confirmed the leaf morphology phenotype as well as the increased GFP expression in the leaf bundles. However, this confirmation was lost in the T_2_ generation, the reason for which could be rearrangements of the inserted T-DNA with loss of expression (Krysan *et al.*, 1999). However, because it occurred independently in both the T_2_ of the *GLDT::NAC052* and of the *GLDT::5'truncatedNAC052*, it is more likely that the *GLDT::(5'truncated)NAC052* transgene post-transcriptionally silences its own protein expression, confirming that NAC052 affects PTGS. The fact that NAC052 plays a role in PTGS should be noted as potentially important to achieve cell-specific gene expression as a first step towards C_4_-like leaf anatomy, as post-transcriptional regulation of photosynthetic genes is a key driver of C_4_ leaf ontogeny (Fankhauser and Aubry, 2017). In addition, the GLDPA gene—the promoter of which was used to create the p*GLDPA*_*Ft*_*::RbcS.TP*-s*GFP* reference line—is known to be subject to nonsense-mediated mRNA decay (NMD), (Wiludda *et al.*, 2012). Both NMD and RNA silencing are part of the post-transcriptional gene silencing process, in which NMD is the front-line RNA quality control pathway, and RNA silencing is induced only when the capacity of the NMD becomes saturated (Christie *et al.*, 2011). The fact that both NAC052 and GLDPA are connected to PTGS and the fact that the *GLDPA* gene was proven to have been important for the establishment of a photorespiratory CO_2_ pump in the genus *Flaveria* (Schulze *et al.*, 2013) suggest that PTGS is essential for the modification of regulatory networks to go from C_3_ photosynthesis towards C_4_ photosynthesis.

**Fig. 6.**
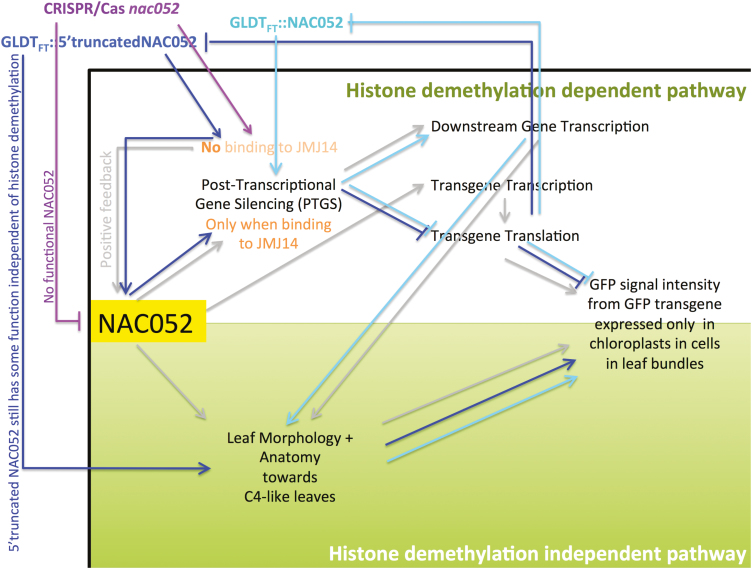
Proposed schematic model of NAC052 function with the effects of introduced *nac052* mutations from this study. The upper half of the scheme in white represents the histone demethylation-dependent pathway for NAC052 function, as described by Ning *et al.* (2015) and by Butel *et al.* (2017); the lower half of the scheme in green represents the histone methylation-independent pathway for NAC052 function, newly described in this study. Grey arrows represent endogenous downstream effects in the NAC052 pathway; dark blue, light blue, and purple arrows represent downstream effects of the genetically introduced *GLDT*_*Ft*_*::5'truncatedNAC052* construct, the *GLDT*_*Ft*_*::NAC052* construct, and the CRISPR/Cas mutation, respectively.

We confirmed the leaf morphology phenotype when mutating NAC052 with CRISPR/Cas. In contrast to the *GLDT::(5'truncated)NAC052* transgenic lines, in the CRISPR/Cas line the leaf morphology phenotype was stable through three generations, suggesting that NAC052 has lost its PTGS effect in the CRISPR/Cas line. Also in contrast to the *GLDT::(5'truncated)NAC052* transgenic lines, in the CRISPR/Cas line the GFP signal observed was significantly lower than in the reference line. Two things can be concluded from these results: the effect on PTGS and the effect on GFP expression caused by mutated NAC052 are linked, but the effect on PTGS/GFP expression and the effect on leaf morphology are not linked. NAC052 is known to be involved in transcriptional repression through both histone demethylation-dependent and demethylation-independent pathways (Ning *et al.*, 2015). The effect of NAC052 on leaf morphology is so far unknown; we propose that the histone demethylation-independent pathway of NAC052 directs the effect on leaf morphology (Fig. 6). The effects of the diverse introduced constructs and mutations in this study on this proposed pathway of NAC052 are drawn in Fig. 6.

In the activation-tagged line, the N-terminally truncated NAC052 leads to enhanced numbers of BS cells around the leaf vein, as well as to enhanced numbers of chloroplasts in these BS cells (Fig. 4). The presence of endogenous NAC052 function in the background of the p*GLDT*_*Ft*_*::5'truncatedNAC052* T_2_ line decreases the effect of the N-terminally truncated NAC052 on chloroplast numbers probably through PTGS (although the number of BS cells is still enhanced, Fig. 4). Together with post-transcriptional silencing of the GFP gene (Fig. 3), this leads to reduced observed GFP signal in the p*GLDT*_*Ft*_*::5'truncatedNAC052* T_2_ line. These results suggest that the increased GFP signal as a result of targeted misexpression of NAC052 after activation tagging comes internally from an increase in chloroplast numbers through the histone demethylation-independent pathway of NAC052 (the histone demethylation-dependent pathway, leading to PTGS, is dysfunctional because of dysfunctional binding between NAC052 and JMJ14). The reduced GFP signal in the p*GLDT*_*Ft*_*::5'truncatedNAC052* T_2_ line comes from PTGS on the GFP transgene. These observations explain how NAC052 functions in leaf development, besides functioning in PTGS.

### Characterization of NAC052 downstream genes

No genes increase/decrease similarly compared with the reference line in the activation-tagged line to in the p*GLDT*_*Ft*_*::5'truncatedNAC052* line, suggesting that the few differentially counted (activated/reduced) transcripts in the activation-tagged line and the p*GLDT*_*Ft*_*::5'truncatedNAC052* line (Fig. 5) come from genes not directly influenced by (5'truncated) *NAC052*. Instead of actively activating/reducing downstream genes, the N-terminally truncated NAC052 only prevents activation/reduction of transcription that otherwise would have taken place following action of the endogenous *NAC052*, confirming the non-functionality of the *5'truncated NAC052* (Table 3).

The transcription of 33 genes was increased in response to endogenous *NAC052* supplemented with bundle-specific expression of (5'truncated) *NAC052*, but not in response to complete knockout of the endogenous *NAC052* (Table 2). These 33 genes were enriched for GO terms ‘cell wall organization and loosening’ and ‘catalytic activity’. A significant part of these 33 genes overlap with up-regulated genes in the *NAC050/052-RNAi* line produced by Ning *et al.* (2015), suggesting that they are downstream genes of endogenous NAC052. More than half of these genes were also differentially transcribed between the total leaf and BS (Aubry *et al.*, 2014), suggesting that these downstream genes of NAC052 play a role in BS metabolism. The N-terminally truncated NAC052 under bundle-specific expression increases transcription of these genes to a lesser extent than misexpressed wild-type NAC052, probably because of the different changes in gene expression (combined gene transcription and post-transcriptional regulation) caused by the interplay of the wild-type and the 5'truncated transcripts of *NAC052*.

Having no endogenous *NAC052* function (CRISPR/Cas line) as well as having bundle-specific misexpression of *NAC052* (p*GLDT*_*Ft*_*::NAC052*) decreases transcription (severely in the CRISPR/Cas line and mildly in p*GLDT*_*Ft*_*::NAC052*) in the leaves of several genes involved in arabinogalactan function as well as one major gene involved in leaf vascular tissue pattern formation (*ALTERED MERISTEM PROGRAM 1*, *AMP1*) and one gene involved in chromatin modification (*VARIANT IN METHYLATION 4*, *VIM4*) (Table 3). Arabinogalactan proteins are a highly diverse class of cell surface glycoproteins, active in the biological processes of cell proliferation and survival, and of pattern formation and growth (Seifert and Roberts, 2007). Arabinogalactan proteins can be considered mediators between the cell wall, the plasma membrane, and the cytoplasm. Many arabinogalactan proteins are glycosylphosphatidylinositol (GPI) anchored, which is a form of post-translational modification common to many cell surface proteins (Seifert and Roberts, 2007). GPI modification serves as a primary plasmodesmal sorting signal (Zavaliev *et al.*, 2016). One arabinogalactan biosynthesis gene (AT4G21060, not identified in this study) has been identified as a candidate gene underlying a quantitative trait locus controlling leaf venation patterning in Arabidopsis (Rishmawi *et al.*, 2017). The other gene with decreased transcription in the p*GLDT*_*Ft*_*::NAC052* line and in the CRISPR/Cas line is *AMP1*, encoding a carboxypeptidase that is known to regulate embryo and meristem development and is linked to leaf vascular tissue pattern formation (Vidaurre *et al.*, 2007). No effects on leaf vein density or venation patterning were found in any line produced in this study. In contrast to the arabinogalactan genes, *AMP1* was also found to be differentially transcribed between the total leaf and BS by Aubry *et al.* (2014), as were four more genes shown in Table 3. The enrichment for expression differences in arabinogalactan genes together with the finding of *AMP1* as a downstream gene of N-terminally truncated *NAC052* in this study strengthens the idea that *NAC052* is a regulator/initiator of leaf developmental changes in Arabidopsis.

### Conclusion

We conclude that targeted misexpression of the transcription factor NAC052 leads to changes in leaf anatomical and morphological development of C_3_*A. thaliana*. This study shows that one of the biological functions of *NAC052* relates to moderating specifically within the leaves the transcription pattern of cell wall organization genes as well as that of arabinogalactan genes, which are mediators between the cell wall, the plasma membrane, and the cytoplasm. As a transcription factor, NAC052 affects many genes, but the effect of *NAC052* on cell wall organization and arabinogalactan genes and their link to leaf anatomical development was so far unknown.

This study was designed to look specifically into the effects of activation tagging on the leaf (pre-) BS cells, in order to find possible regulators to initiate change from C_3_ towards C_4_ photosynthesis. In earlier studies, it was suggested that a pre-existing epigenetic histone code was recruited into the C_4_ promoter control during the evolution of C_4_ metabolism, especially because cell type-specific gene expression patterns in C_4_ species utilize the same functional *cis*-regulatory elements as those in C_3_ species (Heimann *et al.*, 2013; Perduns *et al.*, 2015; Reyna-Llorens *et al.*, 2018). In addition, it was found in maize that genes associated with the C_4_ trait are characterized by a unique class of highly regulated histone marks on upstream promoters (Langdale *et al.*, 1991; Perduns *et al.*, 2015). The fact that NAC052 is a regulator of post-transcriptional gene silencing through histone demethylation as well as a regulator of leaf morphological and anatomical traits that are related to C_4_ photosynthesis supports the suggestion of a histone code being recruited in C_4_ promoter control and provides suggestions for genes that could initiate the first steps believed to activate the C_4_ photosynthetic programme in the BS in C_3_ plants, which is the movement of chloroplasts to the (pre-) BS.

## Supplementary data

Supplementary data are available at *JXB* online.

**Table S1. Primers**

**Fig. S1.** Splice variants for NAC052.

**Fig. S2.** The mutation of NAC052 in the CRISPR/Cas line.

**Fig. S3.** A principal component analysis (PCA) of the RNA sequencing output.

**Fig. S4.** Relative expression in RNA sequencing of the four splice variants of NAC052.

**Fig. S5**. Expression of four splice variants in all three replicates of each line.

Table S1. Primers.

erz509_suppl_Supplementary_Figures
