# Supplementary material for: Targeted misexpression of NAC052, acting in H3K4 demethylation, alters leaf morphological and anatomical traits in Arabidopsis thaliana
Source: J Exp Bot. 2019 Nov 19;71(4):1434–48. doi: 10.1093/jxb/erz509 (PMC7031063; doi:10.1093/jxb/erz509)
Supplement: erz509_suppl_Supplementary_Figures [file erz509_suppl_supplementary_figures.pdf]

**SUPPLEMENTARY FIGURES** van Rooijen et al.

Targeted misexpression of *NAC052*, a H3K4 demethylase, alters leaf morphological and anatomical traits in *Arabidopsis thaliana*.

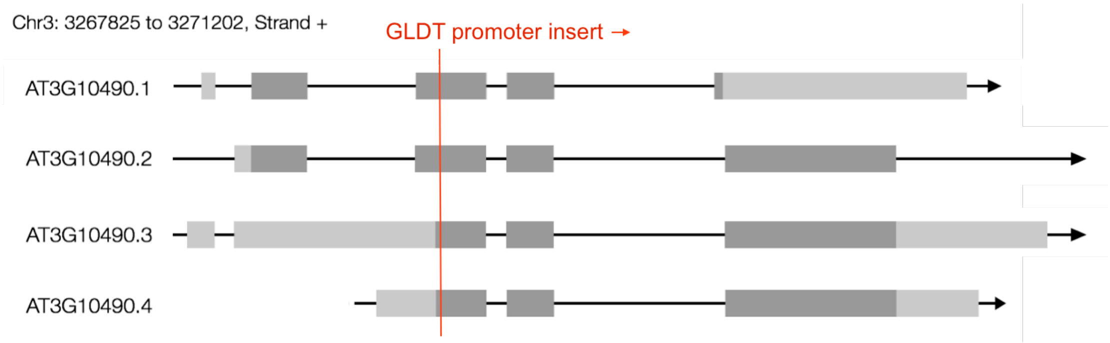


**Supplemental Figure S1. Splice variants for *NAC052***

Dark grey represent coding sequence (CDS), light grey represents untranslated region (UTR), black line represent introns.

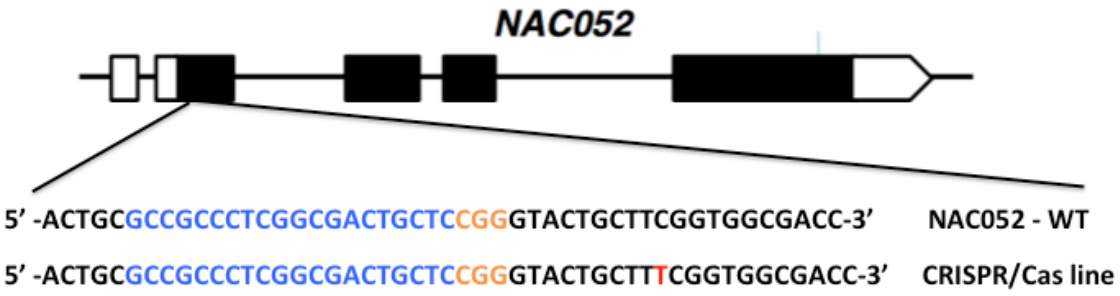


**Supplemental Figure S2. The mutation of NAC052 in the CRISPR/Cas line**

Sequence in blue represents the sgRNA targeting sequence. The protospacer adjacent motif (PAM) required for sgRNA recognition is in orange. In the CRISPR/Cas line created in this study, a thymine nucleotide indicated in red is inserted and leads to a frame shift.

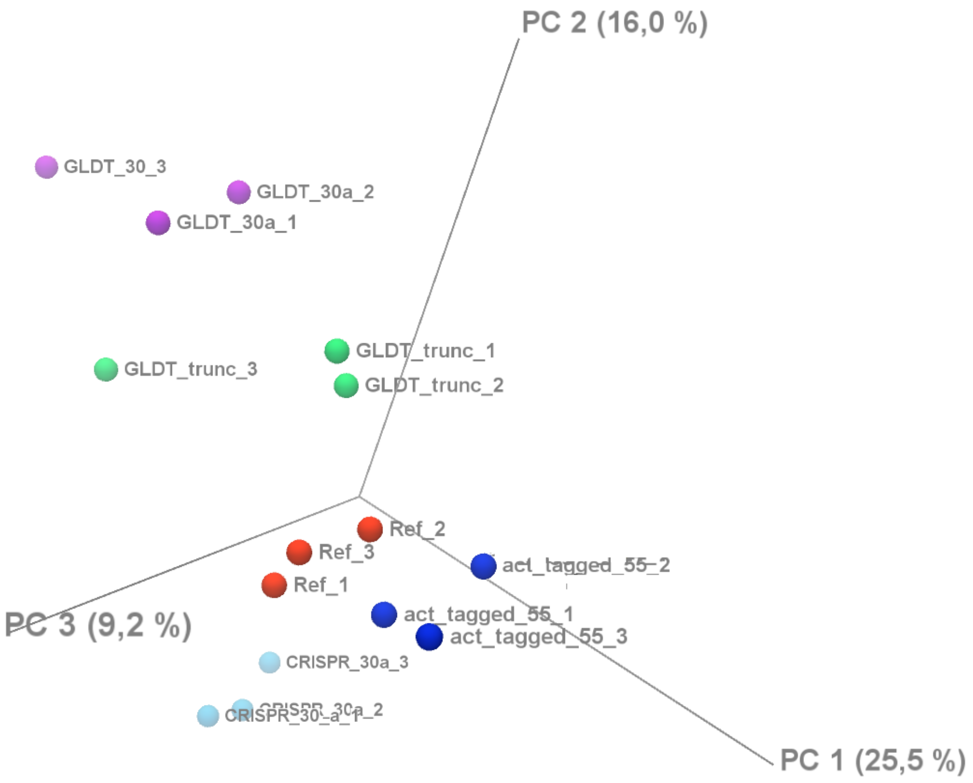


**Supplemental Figure S3. A principal component analysis (PCA) of the RNA sequencing output**

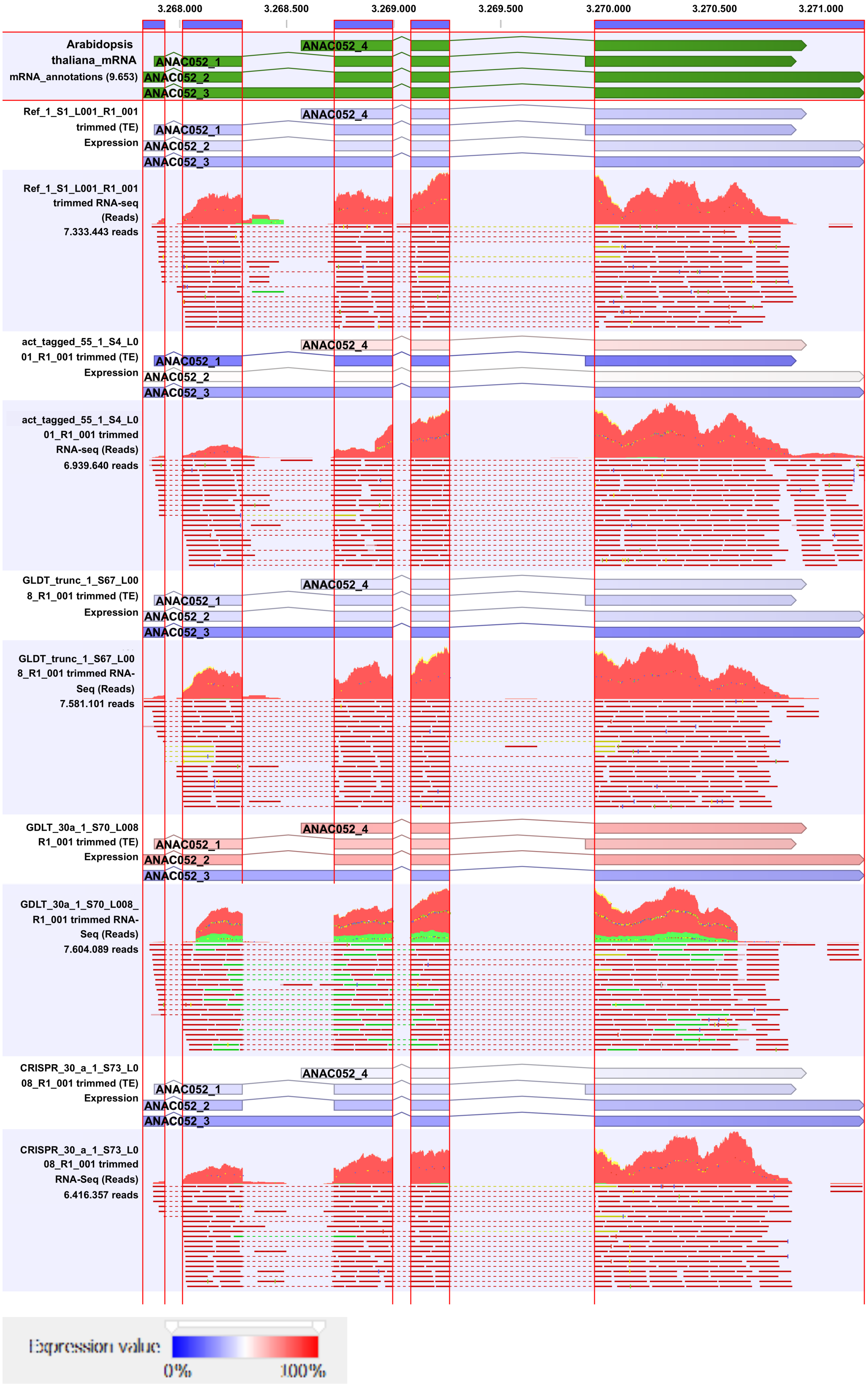


**Supplemental Figure S4. Relative expression in RNA seq of the four splice variants of NAC052.**

The gene structure of the 4 splice variants is shown in green on top of the figure, followed by the same gene structure of the 4 splice variants in either red or blue representing the expression value of the splice variants, followed by mapping the reads. Each line is represented by one replica

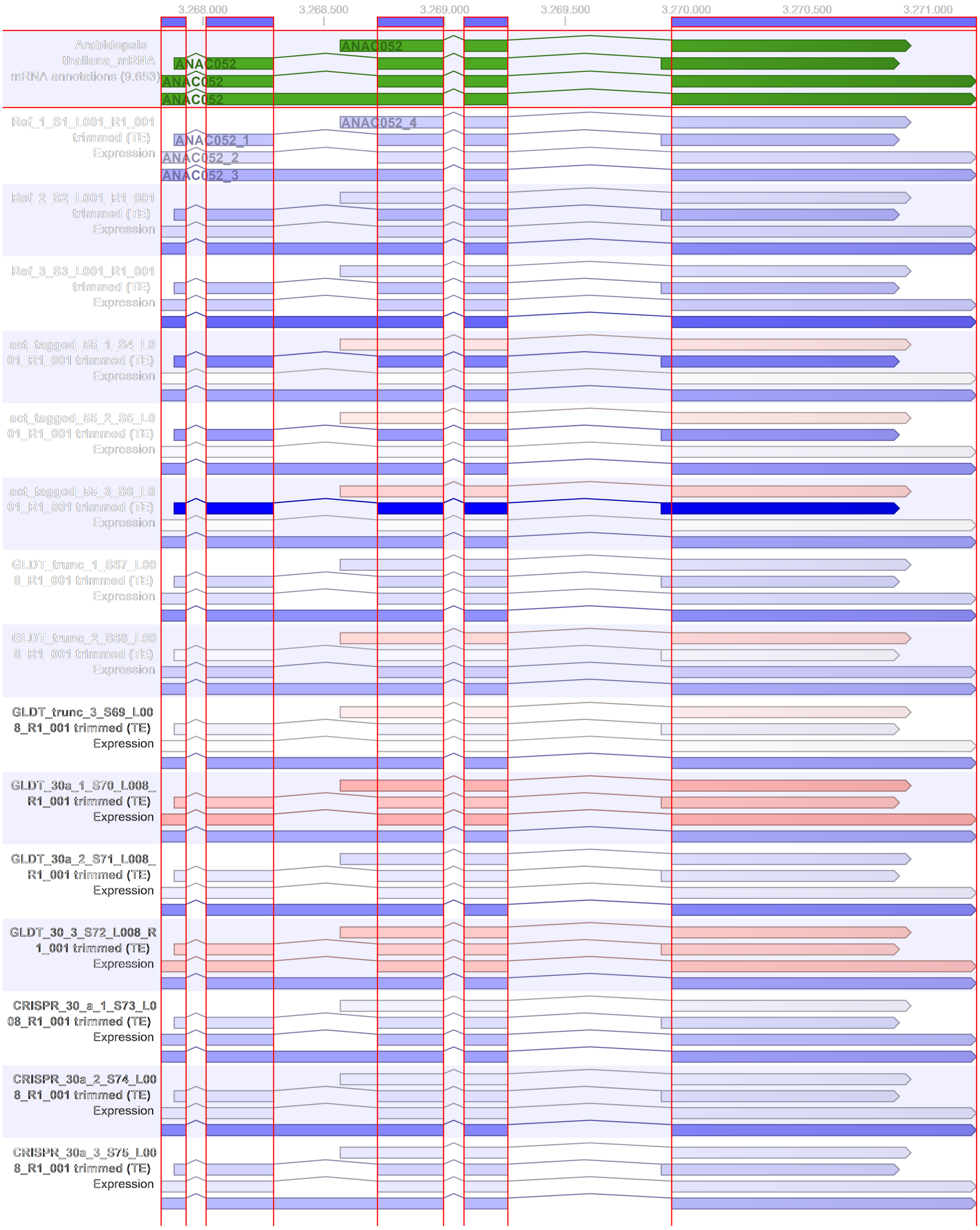


**Supplemental Figure S5. Expression of four splice variants in all three replica's of each line.**
